# Total T4 rise in pregnancy: a relook?

**DOI:** 10.1186/s13044-020-00088-5

**Published:** 2020-07-31

**Authors:** Subhodip Pramanik, Pradip Mukhopadhyay, Sujoy Ghosh

**Affiliations:** Department of Endocrinology and Metabolism, I.P.G.M.E&R, Kolkata, West Bengal 700020 India

**Keywords:** Thyroid function tests, Pregnancy, Reference range, Total T4

## Abstract

**Background:**

Total T4 (TT4) measurement is preferred to free T4 (FT4) especially in last part of pregnancy. Guidelines by American Thyroid Association, European Thyroid Association and Endocrine Society state that TT4 increases 1.5 times pre-pregnant levels after week 16 of pregnancy. However, this is based on a small study conducted 40 years ago which used radioimmunoassay for determination in changes in TT4.

**Materials and methods:**

A cross-sectional study was undertaken to find reference interval for thyroid function in different trimester of pregnancy with special reference to look at the degree of elevation of TT4 as compared to non-pregnant women. Two hundred non-pregnant women (excluding oral contraceptive users) and 600 pregnant women (200 from each trimester) aged 18–40 years were consecutively recruited starting from around 6th week of pregnancy having confirmed singleton pregnancy diagnosed at 8th week by ultrasound. The exclusion criteria included: (1) a personal or family history of thyroid disease; (2) presence of goiter or nodule confirmed by ultrasound; (3) anti-TPO antibody positive state (titre > 35 IU/ml). All subjects were tested for urinary spot iodine concentration and those with UIC < 150 μg/L were excluded. Finally, thyroid function tests (TSH, FT4, TT4, TT3) of 168 non-pregnant women and 163, 153 and 148 women at 1st, 2nd and 3rd trimester respectively were analysed..

**Results:**

Total T4 (mean ± SD, μg/dl) in non pregnant women and in different trimesters was 8.95 ± 1.71, 9.71 ± 2.39, 12.11 ± 1.55, 11.83 ± 1.49 respectively. Rise in TT4 occurred between 10-18th week. The mean TT4 in second trimester increased by 25% as compared with the value at 6-9th week and by 35% as compared to non-pregnant value.

**Conclusion:**

Rise in total T4 in second trimester pregnancy is only around 25% as compared to first trimester value and 35% than the non-pregnant value. Hence multiplying non-pregnant T4 value by 1.5 may actually over-diagnose maternal hypothroxinemia and lead to inappropriate diagnosis and treatment of isolated maternal hypothyroxinemia in a significant proportion of subjects.

## Background

Non-pregnant cut-offs for thyroid function do not appropriately reflect thyroid status during pregnancy. Current guideline suggests, instead of measuring FT4 (free T4), measurement of TT4 (total T4) (with a pregnancy-adjusted reference range) is a highly reliable means of estimating hormone levels during last part of pregnancy [1]. FT4 assay during pregnancy is highly variable and depends on assay method and age of gestation [1, 2]. Koevaar et al. suggested that maternal TT4 levels, especially in first half of pregnancy are either not associated or not better associated as compared to FT4, with adverse pregnancy or child outcomes [3].

American Thyroid Association, European Thyroid Association and Endocrine Society suggests that we use TT4 during pregnancy to diagnose hypothyroxinaemia and normative reference ranges in pregnancy for TT4 is determined by multiplying the non-pregnant value by 1.5 in second and third trimester [1, 4, 5]. Total T3 and T4 starts to increase gradually and peaks by 16th week of pregnancy and the magnitude of increase is stated to be upto 50% of that at the start of pregnancy. If a T4 measurement is required in the early part of pregnancy (i.e., weeks 7–16 of pregnancy), it is suggested that we can estimate an upper reference range based on increasing the non-pregnant upper reference limit by 5% per week (starting from 7th week of gestation). The assumption that there is a 50% rise in T4 is based on a study by Weeke J et al. which documented that T3 and T4 are approximately 1.5 times of the values measured 10 weeks post-partum [6]. However this study was conducted in 1982 and the values were documented longitudinally from the same cohort of women upto 12 wks after delivery. The study had several limitations, namely, small sample size (only 20), subjects were not evaluated for possible underlying subtle thyroid diseases (including presence/absence of goiter, autoimmunity). Additionally iodine status was not evaluated and an older generation of radioimmunoassay was used. In this study it is assumed that thyroid function test at 12 weeks postpartum is representative of pre-pregnant values. This assumption may be fallacious due to high prevalence of postpartum thyroiditis (upto 22% in some studies) [7].

In the study by Zhang et al., when the lower limit of non-pregnant TT4 is multiplied by a factor of 1.5; it was found that almost 29.3% of subjects in first trimester of pregnancy would fulfil the diagnostic criterion of hypothyroxinemia [8]. This obviously would over-diagnose hypothyroidism in pregnancy as compared to other studies [9] and possibly result in inappropriate levothyroxine therapy in a significant proportion of pregnant women. Korevaar et al. also demonstrated that the change of mean maternal TT4 levels was 27.5% higher at 18th week of pregnancy as compared to 8th week [3].

Considering all of the aforementioned limitations and conflicting data and with availability of newer assay methods, it is imperative to have a relook at the quantum of change of T4 during pregnancy.

## Main text

We undertook a cross-sectional study to establish reference interval/normative data for thyroid function tests in an Indian population with special reference to determine the quantum of change of total T4 during pregnancy as compared to non-pregnant women. The preliminary data of the on-going study has been published [10]. The data presented herein is an extension of the said study with inclusion of more subjects.

Two hundred non-pregnant women (excluding oral contraceptive users) and 600 pregnant women (200 from each trimester) aged 18–40 years were consecutively recruited starting from around 6th week of pregnancy having confirmed singleton pregnancy diagnosed at 8th week by ultrasound. The exclusion criteria included: (1) a personal or family history of thyroid disease; (2) presence of goiter or nodule confirmed by ultrasound; (3) anti-TPO antibody positive state (titre > 35 IU/ml). All subjects were tested for urinary spot iodine concentration and those with UIC (Urinary Iodine Concentration) < 150 μg/L were excluded. After exclusion, the number of subjects eligible in 1st, 2nd, 3rd trimesters and non pregnant group was 163, 153, 148 and 168 respectively.

TT4 was estimated by Chemiluminescence technique (CLIA) using commercially available kits from Siemens Diagnostics (Germany) with Immulite-1000 analyzer. The analytical sensitivity was 0·4 μg/dl. The laboratory reference ranges was 4.5–12 μg/dl and the inter-assay coefficients of variation (CV) was 6.7%.

**Fig. 1:**
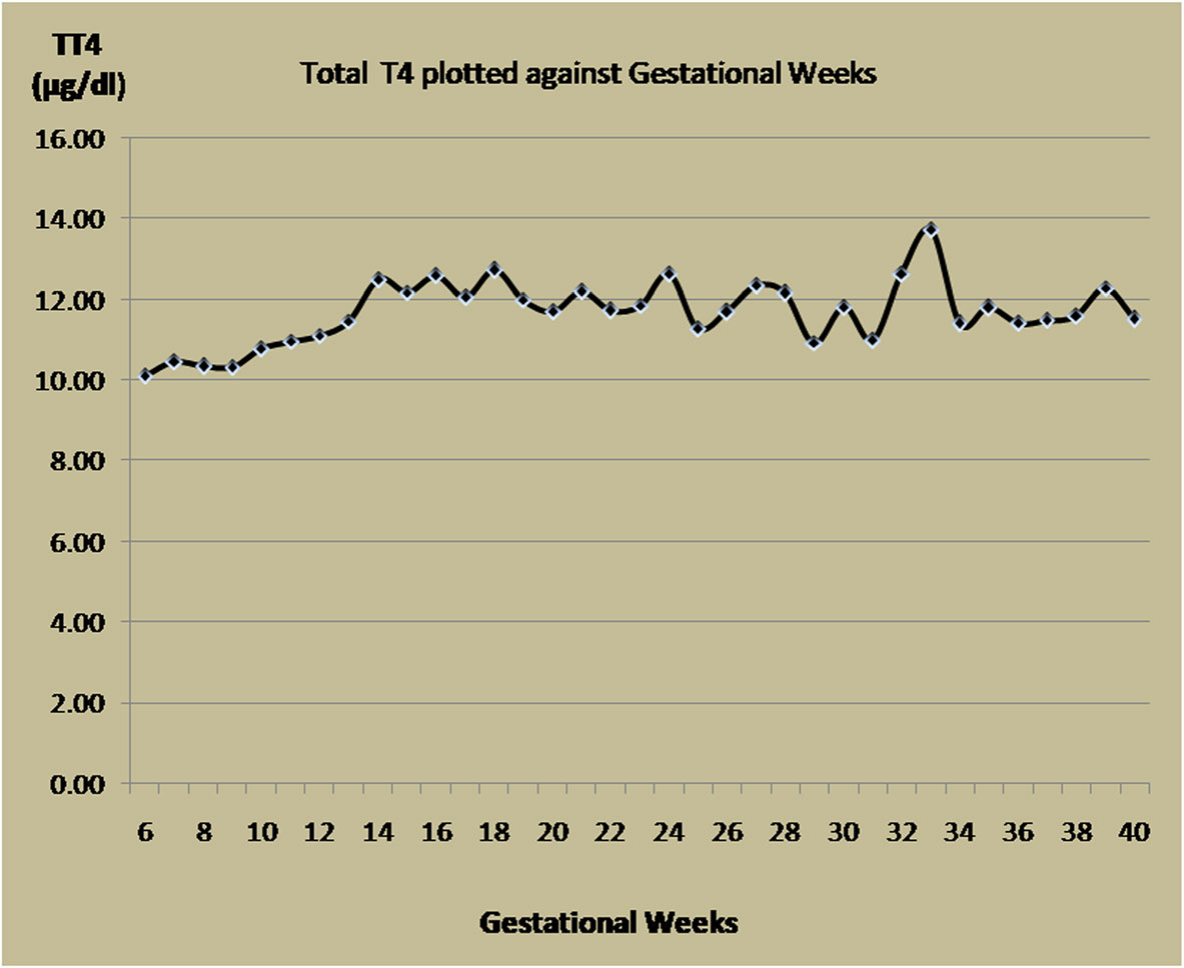
Total T4 plotted against gestational weeks

Total T4 (mean ± SD, μg/dl) in non-pregnant women and in different trimester was 8.95 ± 1.71, 9.71 ± 2.39, 12.11 ± 1.55, 11.83 ± 1.49 respectively. TT4 increased from around 10–12 week to reach a peak around 18th weeks, after which it almost plateaued. The increment at 10–18 weeks is approximately 25% (24.71%) as compared to 1st trimester values (Fig. 1). Compared to the non-pregnant women, the increment in TT4 is approximately 35% (35.30%).

Zhang et al. compared the relationship between self-sequential longitudinal levels of thyroid hormones with cross-sectional levels, while investigating the reference range of thyroid function during pregnancy [8]. They established that there is no significant difference between a self-sequential longitudinal reference interval and a cross-sectional reference interval [8]. Hence we used a cross-sectional reference interval in our analysis for convenience. In most situations, pre-pregnancy thyroid function test reports are not available. Hence we tried to document the degree of elevation of TT4 with respect to first trimester value also. A possible limitation of the study is that we evaluated anti-TPO antibody, but did not check anti-thyroglobulin antibody.

The finding of maximum increment in TT4 by 35% with respect to the non-pregnant women is in clear contrast with the traditional wisdom that TT4 rises maximally upto 50% of the non pregnant value after week 16 of pregnancy [1]. This finding has significant clinical implications and should be validated in larger cohort and in other ethnic groups.

## Conclusion

We conclude that the rise of TT4 peaks between 10-18th week of gestation and mean TT4 during second trimester is increased by 25% as compared with 6-9th week and by 35% as compared to non-pregnant value which is much less than what current guidelines suggest. This we believe would lead to avoidance of inappropriate diagnosis and treatment of isolated maternal hypothyroxinemia in a significant proportion of subjects. This finding also reinforces the necessity of establishing trimester-specific hormone ranges to diagnose thyroid dysfunction during pregnancy.

## Data Availability

Available

## Abbreviations

TT4: Total T4
FT4: Free T4
UIC: Urinary Iodine Concentration
TPO: Thyroid peroxidase
